# Does Reality Overcome the Expected? Survey on Informal Caregivers’ Profile: A Nurse-Led Study in Times of COVID-19

**DOI:** 10.3390/ijerph191811394

**Published:** 2022-09-10

**Authors:** Maria Adriana Henriques, David de Sousa Loura, Paulo Nogueira, Graça Melo, Idalina Gomes, Isabel Ferraz, Laura Viegas, Andreia Costa

**Affiliations:** 1 Nursing Research, Innovation and Development Centre of Lisbon (CIDNUR), Nursing School of Lisbon (ESEL), 1600-096 Lisbon, Portugal; 2 Instituto de Saúde Ambiental (ISAMB), Faculdade de Medicina, Universidade de Lisboa, 1649-028 Lisbon, Portugal; 3 Nursing School of Lisbon, 1600-096 Lisbon, Portugal; 4 Hospital Dona Estefânia, Centro Hospitalar Universitário de Lisboa Central, E.P.E., 1169-045 Lisbon, Portugal; 5 Laboratório de Biomatemática, Instituto de Medicina Preventiva e Saúde Pública, Faculdade de Medicina, Universidade de Lisboa, Avenida Egas Moniz, 1649-028 Lisbon, Portugal

**Keywords:** informal caregivers, nursing, survey, health profile, community

## Abstract

The increasing overload of chronic conditions raises challenges for the health system. Informal caregivers have a major role in ensuring the quality of life of the cared-for person, despite the reported lack of working resources which can lead to unmet needs. This article reports on the first part of a nurse-led research project entitled ‘‘Informal caregiver’ profiles in Lisbon county: a health community approach.’ We aimed to support decision-making by developing an informal caregiver profile to promote tailored interventions. A survey addressing the dyad was developed and submitted to a convenient, network-based, stratified sample of carers aged 18 years or above. More than thirty community partners supported the identification of caregivers. Data were submitted to univariate descriptive analysis. A profile of the cared-for person and the informal caregiver was uncovered by identifying 639 caregivers, of whom the majority lived with the cared-for person. Only four percent planned the transition to a caregiver role, and no more than 10% had access to support programs. Approximately half of the respondents found that COVID-19 negatively impacted their performance in the caregiver role. Developing a local and tailored strategy with collaboration between healthcare professionals, academics, and community partners is key to ensuring that meaningful support is provided to caregivers.

## 1. Introduction

Aging demographics worldwide and the consequent rise in life expectancy have led to a new population profile, in which the geriatric population has increased [1]. The situation at the European level is coherent with these data as approximately one-fifth of the population is above 65 years old, presenting an average life expectancy of 20 years, in which only ten are disease-free [2]. Portugal is not the exception, as the aging rate has grown by 44% from 2010 until 2020 [3], a higher variation than in other Mediterranean countries such as Spain, Greece, and Italy, where this percentage ranged from 21% to 25% [4]. In Lisbon, there were only two active individuals per senior citizen in 2020 [5].

It is known that the probability of having a chronic condition increases with age [2,6]. Considering the present situation regarding life expectancy and the existing tools for health promotion and disease prevention at our disposal [1], living with a chronic condition has become more frequent. Furthermore, UNICEF emphasizes that the youth population has also been experiencing an increase in disease overload: although a ratio of 1 child with disability per 10 children worldwide is reported [7], in adolescents, most of the burden is related to behavioral problems requiring intervention targeting lifestyle modifications [8].

According to the NCD Alliance, one-fifth of the world’s population has been diagnosed with an NCD, and multi-morbidity is a growing trend [9]. Therefore, considering that people with chronic diseases could become potential recipients of care, whether formal or informal, it is important to assess and reflect on the available resources to provide long-term care and consider the central role of informal or family caregivers and communities in its implementation and maintenance [10]. Published data show that it is unlikely for caregivers to exist in an adequate number to provide care to all persons in need of support by 2060 [11].

According to the OECD, informal or family caregivers are “(…) people providing any help to older family members, friends, and people in their social network, living inside or outside their household, who require help with everyday tasks” (p. 234) [12]. The caregiver can be a family member, partner, friend, or neighbor, assisting someone with whom they have a personal and meaningful relationship [13]. A person in need of care or care receiver/recipient is defined as a person with a chronic condition or disability who needs help to perform basic and instrumental daily living activities, as well as emotional support and assistance with managing their care routine [14].

In 2021, there were approximately 71 million caregivers across the European Union (EU) [15], nearly double the formal caregiver force at a global level, accounting for more than 75% of long-term care in the EU [16]. At an economic level, the work of informal caregivers is equivalent to at least 2.5% of the European GDP, with more than 33 billion hours being spent per year on this activity [17]. In Portugal, 12.5% of the population was identified as a caregiver [15], which is in line with the European range (12% to 18%) [14].

Becoming a caregiver is complex, as it can be challenging physically, psychologically, socially, and spiritually [18]. This process can be planned and gradual, although it is frequently experienced as a sudden change, particularly in the case of early discharges from the hospital, where there is little or no time to prepare caregivers for the tasks they will need to be able to perform [19].

Distress generated by caring for a highly dependent person can be increased by these aspects and have a negative influence on the quality of life, personal management, and professional performance of the caregiver [19,20,21,22]. Inefficient management of these situations and the lack of resources to support caregivers can lead to unattended needs and caregiver burdens [23]. Therefore, the health of the caregiver has to be a priority when talking about long-term care, given the need to prevent feelings of burden and overload, as well as to ensure the quality of life of informal carers and cared-for people.

This perspective is in line with the Model of Carer Stress and Burden [24], which affirms that the outcomes of caregiving can be explained by the interaction between background and contextual factors (sociodemographic variables); primary stressors (related to the caregiving activity itself, particularly regarding patient characteristics, the care situation, and carer needs); secondary stressors (regarding the context of caregiving activities and articulation with social, financial, and personal variables); appraisal (the caregiver’s subjective evaluation of capability and control, as well as the availability of resources); and moderators (exacerbating and moderating factors, such as knowledge, competence, coping resources, caring background, formal service use, among others). As a complex phenomenon, informal caregiving must be addressed as a process, generating interventions to tackle problems in each one of these dimensions.

Although some countries have invested in identifying caregivers and their needs, much work remains to be completed. The literature describes a major deficit in networks for the identification of caregivers [16] and highlights that most governments consider caregivers’ needs as an appendix to the ones identified for the cared-for person [6]. Furthermore, the Directorate-General for Employment, Social Affairs, and Inclusion of the European Commission and ECORYS, the authors of a study on the incidence and costs of informal long-term care in the EU, indicate that long-term care systems are different across Europe, with particular concerns about their development in southern and eastern regions [14]. According to Spasova et al., the inexistence of a large formal caregiver workforce, low-quality standards, and the high cost of the few existing long-term care facilities, as well as the tradition of caring within the family, are substantial contributing factors to these differences [17].

In Portugal, the importance of informal caregivers has been highlighted by the publication of the Informal Caregiver Statute in 2019, a law designed to define the role of caregivers and care receivers, outlining their rights and duties and providing guidelines on measures to support them. However, the mechanisms described in the document, such as negotiating between professional activities and caregiving, caregiver rest services, and financial support, are in a pilot stage and are yet to be fully implemented [25].

The needs of caregivers across Europe are multiple and have been studied throughout the years. In 2005, the EUROFAMCARE project provided a trans-European profile of informal caregivers’ demographic characteristics and needs in six countries, concluding that more services for informal carers and cared-for people were needed, together with regular needs assessments, integrated care training, and other practices [26]. More recently, systemic measures such as improving financial investments to support long-term care systems [6], the involvement of caregivers in the diagnosis of needs and the development of caregiver-related laws [21], and global awareness campaigns targeting the importance of informal caregivers [27,28] have been identified as crucial requirements in facing the rise of informal caregiving.

The COVID-19 pandemic has increased the number of caregivers and has introduced more challenges for them. A study from Eurocarers and the National Institute of Health and Science of Ageing—Italy (IRCCS-INRCA) indicated that, because of this sanitary crisis, the number of caregivers increased in Europe: approximately 12% started their caregiving role because of COVID-19 [27]. In Portugal, about 25% of participant caregivers reported that they had become carers following a situation related to COVID-19 [27].

An acknowledgment of the problems and deficits targeting informal caregivers motivated the present study, recognizing that the need for the identification of caregivers and exploration of the issues they experience is critical in the city of Lisbon. As a fundamental part of the health system, nurses are required to provide individualized care with attention to the details of each specific individual, family, or community [29]. Therefore, developing research on informal caregivers, especially indicators linked to the role of nurses, is recommended by the European Federation of Nurses Associations as a starting point to inform better and individualized policy decisions [30]. Following a health community approach, in this study, we aimed to (1) develop a profile of Lisbon’s informal caregivers; (2) inspire the adoption of tailored interventions based on this study’s results, designed to support decision-making regarding informal caregivers at a local level. There was no a priori hypothesis defined.

## 2. Materials and Methods

The reported methodology was based on the Checklist for the Reporting of Survey Studies (CROSS) [31]. Sections according to this instrument are presented as follows.

### 2.1. Study Design

This study is part of a project with a multistudy and multimethod approach [32], which is being developed through two stages. In the first stage, a mixed-methods study and a qualitative study were developed. This paper reports a mixed-methods study with a descriptive cross-sectional design.

### 2.2. Data Collection

A data collection survey was developed by the authors, including the following sections: provenience; information about the person receiving care; information about the caregiver, and evaluation scales regarding the caregiver. The form included questions related to both the caregiver and the cared-for person regarding sociodemographic information, family relationships, caregiving facilities, the health of the dyad, and the prevalence of chronic conditions or comorbidities.

Measurement instruments to assess the level of dependence in the activities of daily living in the cared-for person were also applied. All instruments were presented in Portuguese.

The Lawton and Brody Instrumental Activities of Daily Living Scale [33,34] was included to evaluate care recipients’ dependence on instrumental activities of daily living, such as their capability to use the telephone, go shopping or manage economic matters, among others. The capability to perform such activities was rated individually, with options referring to the level of limitation, blindly rated from zero (0—high dependency) to one (1—partial dependency or independent). The final score was determined through the sum of these rates, corresponding to a level ranging from high dependence to independent. This instrument has been validated for the Portuguese population and its psychometric properties are appropriate for use in this study, presenting a satisfying reliability (α = 0.90) and the inexistence of redundancy (0.52 < *r* < 0.80) [34].

The Barthel Index for Activities of Daily Living [35,36] was used to assess dependence in satisfying basic daily needs, such as the capability to take care of personal hygiene, eat, and climb stairs, among others. This assessment was carried out through the selection of the option that better described the limitations of the individual in each item of the scale, which corresponded to an individual classification ranging from total dependency to independency. The final score was calculated through the sum of all individual classifications, graded from total dependency to total independency. This instrument has also been validated for the Portuguese population with adequate psychometric properties, particularly high reliability (α = 0.96) and adjusted redundancy (0.66 < *r* < 0,93), similar to other studies [36].

The survey also included several open-answer questions, allowing the participants to describe better topics such as housing and accessibility conditions, perspectives on the rights and duties of the caregiver, and opinions about possible additions to the Statute of the Informal Caregiver.

Before the publication of the data collection survey for caregivers’ participation, the instrument was submitted for internal review by the research team to identify inaccurate questions or to optimize the wording. An internal pilot testing stage undertaken by members of the research team and a group of collaborators, who were not responsible for the development of the questionnaire, was also undertaken. These processes resulted in the optimization of the questionnaire regarding its presentation and content. The possibility of participating in the qualitative study of the project’s first phase was also offered through e-mail address insertion.

### 2.3. Sample Characteristics

A convenient, network-based, and stratified sample was considered [37]. Inclusion and exclusion criteria were applied as described in Table 1. Institutional partnerships with local and national structures (‘partners’), such as primary health care centers, NGOs, and the municipality, were developed to cooperate in the identification and selection of eligible caregivers and cared-for people to whom care was provided in the community, with these partnerships providing the basis for sample stratification.

All caregivers were identified by partners, who made the first contact, assessing the dyad’s availability to participate in the research study. If the response was positive, communication between a project collaborator, the caregiver, and the cared-for person was established to expedite survey administration. The research team had no role in selecting caregivers prior to the partners’ first contact. All referred caregivers were contacted.

### 2.4. Survey Administration

Data collection was open between March and September 2021. The survey was available online (Limesurvey^®^ platform) and on paper. The physical questionnaire was applied in a home visit context by nurses working in the community. 

Following the acceptance of the dyad to be a part of our study, a second contact was performed by a project collaborator, aiming to explain how to participate in the study: via s direct interview, in which a project collaborator would conduct a live interview via telephone, or an autonomous response, in which the survey was answered by the caregiver and the cared-for person without direct support, and a touch-base meeting was scheduled to evaluate possible doubts or to confirm the response. This information was registered on a shared document, only accessible to the research team and interviewers. Individual follow-up by the project collaborators was promoted, aiming to prevent multiple participation and biased results. The caregivers and care receivers were not paid to participate in the study.

In this process, 16 undergraduate students in Nursing and nine postgraduate students in Community Nursing and Medical and Surgical Geriatric Nursing collaborated as interviewers under the supervision of a project collaborator. Periodically, an incomplete answer report was extracted from the digital platform, and an e-mail was sent (when available) to these participants to remind them to complete the survey and state the project team’s availability to provide support.

### 2.5. Study Preparation

The research team included several nursing researchers and professors linked to a higher nursing school and two nursing research collaborators. The research team organized several meetings to prepare the survey administration and discuss the data collection form and the strategies to enhance the dissemination of the survey. A procedure for administering the survey was developed to help the interviewers in their tasks, as well as a private direct communication channel with the project supervision team to promote support and clarification if needed.

When the survey was administered, a dissemination strategy was implemented to boost responses, including social media publications and collaboration of project partners, urging their networks to fill out the survey.

### 2.6. Statistical Analysis

Data were saved and archived in the Limesurvey^®^’s back office. All responses until the section “Information about the caregiver” were accepted as valid, whether globally complete or incomplete (if incomplete, the particular answer was stated as Non-Applicable or Non-Answered). All other responses were discarded.

Microsoft Excel^®^ and IBM^®^ SPSS^®^ Statistics were used for descriptive univariate statistical analysis. A codification manual was developed to assist in the process. 

Concerning the open-answer questions, content analysis was conducted [38]. For the majority of questions, a posteriori classification was the chosen approach, in which context units were defined from parts of the text to extract significance and, therefore, codify classifications. For inquiries related to the rights and duties of the caregiver, a priori categorization was undertaken, given that the Statute of the Informal Caregiver was considered as a reference for defining context units and the text was analyzed through that lens.

### 2.7. Ethical Considerations

The project, including the phase reported in this study, was submitted to the Ethics Committee for Health of Lisbon and Tejo Valley’s Regional Health Administration (Process number 105/CES/INV/2020) with a declaration of approval before the start of data collection, in February 2021.

All ethical procedures were completed. All participants accepted the participation conditions through the signature of two free, prior, and informed consents—one for the caregiver and one for the cared-for person. Data collection was conducted using an untraceable and exclusive coding system for each response, ensuring participant anonymity and data confidentiality. Only the principal investigator and the research collaborator responsible for statistical analysis had access to the back office of the survey. All the researchers and collaborators involved filled out a confidentiality agreement.

## 3. Results

### 3.1. Respondent Characteristics

Data collection was performed between March 2021 and September 2021, with 639 caregivers indicated by partners. Approximately 28% (n = 179) of these caregivers did not participate in the study, due to the lack of an answer to the phone call (n = 131, 21%); unavailability/no interest in participation (n = 40, 6%); lack of identification as a caregiver, institutionalization of the cared-for person, and a lack of health conditions to participate (n = 8, 1%).

Therefore, 460 caregivers indicated by partners were included in our study, with the addition of 71 caregivers who had access to the questionnaire by other means. A 64% response rate was reported (343 caregivers answered the whole questionnaire or up to the section on the information about the caregiver). No sociodemographic differences were found between participants and caregivers who refused to participate or did not complete the questionnaire.

Most caregivers provided care to one person, although 11% (n = 37) indicated that they were responsible for caring for more than one person.

### 3.2. Generic Information about the Person in Need of Care

The cared-for people included in this study were mostly women (n = 230, 72%) aged between 2 and 102 years old, even though 75% were over 76 years old (*M* = 80.3; *SD* = 17.2). Their marital status was predominantly widowed (n = 144, 45%) and the majority had concluded primary education (n = 129, 40%).

Eight out of ten care recipients received care from their informal or family caregiver for over a year, and half had already been in this condition for six years or longer. For most cared-for persons, disease was the main reason causing the dependence (n = 192, 56%). Following a classification developed in the WHO International Classification of Diseases and Related Problems in its 10th version (ICD-10), mental and behavioral disorders (n = 38, 22%) appeared to be the most frequent disease-causing dependence with a need for care (these included dementia and personality disorders), followed by diseases of the circulatory system (n = 35, 20%), such as strokes and myocardial infarctation, and diseases of the nervous system (n = 34, 19%), such as Parkinson’s and Alzheimer’s diseases. Seven out of ten persons had two or three comorbidities.

Two measurement instruments were applied to assess the level of dependence of the cared-for persons. The Barthel Index for Activities of Daily Living [25,26] showed that approximately one-third of the cared-for persons (n = 113) exhibited total dependency, and 19% (n = 65) exhibited severe dependency in their daily living activities. Furthermore, the results of the application of the Lawton and Brody Instrumental Activities of Daily Living Scale [23,24] demonstrated total dependency in the instrumental activities of daily living in more than two-thirds of the sample (n = 228). Only 14% (n = 47) displayed a moderate or slight dependence.

### 3.3. Sociodemographic Information about the Informal/Family Caregiver

Informal or family caregivers in Lisbon were mostly women (n = 231, 73%) aged between 22 and 94 years old (*M* = 62.3; *SD* = 13.1). Most caregivers were married (n = 167, 57%), and almost half (n = 131, 44%) had completed at least one higher education level. Concerning their social situation, most respondents were retired (n = 120, 43%), and 18% retired to care for a person in need. Employed caregivers worked in several fields, the most common being intellectual and scientific activities (n = 85, 29%), followed by administrative personnel (n = 71, 24%). Almost 40% (n = 113) of the caregivers received a monthly salary between 665 and 1270 euros.

### 3.4. Context of Caregiving

Concerning the physical space where care is given, caregivers identified that these were generally in good housing conditions. However, 14% described problems related to dimensions (n = 31), maintenance (n = 8), or the absence of necessary infrastructures (n = 8). Accessibility was also analyzed, and a significant percentage of carers reported impaired conditions related to this matter: 20% (n = 77) reported problems in the house exterior and 10% (n = 34) in the interior. 

More than two-thirds of caregivers lived with the person whom they were caring for regularly (n = 195, 64%) or occasionally (n = 16, 5%). The average time required to reach the caregiving facility when the caregiver and the cared-for person did not live together was 33 min. 

Our study indicated that daughters (n = 123, 39%) were the most common carers, whereas only 26% (n = 81) were a spouse. Planning the process of becoming a caregiver was only a reality for 13 carers in our sample (4%). Approximately, 35% (n = 104) suddenly assumed this function and the majority became a carer through a progressive process (n = 179, 61%).

Seven out of ten carers identified love/affection as the main reason for caregiving (n = 204). They wished to maintain the presence of the cared-for person at the house (n = 139, 46%) and a feeling of duty and obligation in caring (n = 134, 44%) and the belief that the cared-for person would do the same if the roles were reversed (n = 115, 38%) were other reasons mentioned.

Nearly 83% (n = 283) of the caregivers provided care for more than a year (*M* = 7.6; *SD* = 7.4); those who did not supported the cared-for person for five months on average (*SD* = 2.7). Per day, these people dedicated, on average, 12 h (*SD* = 9.2) to providing care, although about one quarter (n = 86) performed these activities 24 h a day.

### 3.5. Care provided by the Caregiver to the Cared-for Person

Concerning care provided by the caregiver, the areas of daily living activities, emotional support, and care coordination were assessed.

Support for basic daily living activities (Table 2) was more significant for dressing (56%), the choice of clothes (53%), and feeding supervision (53%). In regard to instrumental daily living activities (Table 3), shopping (88%), managing finances and legal matters (81%), and going to clinical appointments with the cared-for person (78%) were the most mentioned assistance activities.

Emotional support and helping people in need to feel safe were also identified as pivotal activities in a caregiving role by more than 75% (n = 236) of the carers. Approximately six out of ten (n = 193, 56%) provided specific support for managing complex behaviors, such as sadness or aggressiveness. 

Comparatively, care coordination and management were also described by caregivers as their responsibility, particularly seeking support services (n = 175, 58%), supervising formal care (n = 117, 39%), and communicating with their family (n = 106, 35%).

A great majority of the respondents considered that they had help from other people at a necessary/sufficient level (n = 138, 47%) to provide support, despite more than one-third revealing that they had less help than needed (n = 103, 35%) or a total lack of help (n = 31, 11%).

### 3.6. Support from Family or Friends in Care Provision by the Caregiver

According to the collected data, almost half of caregivers (n = 138, 47%) benefitted from the support of family or friends to provide care to a person in need. Of those, one-third received the help of a son/daughter (n = 44, 34%), followed by spouses (n = 19, 15%) and parents (n = 19, 15%). Only about 20% (n = 28) of the respondents counted on more than one person performing this activity.

Twenty-three hours per week was the average time dedicated by a family member or a friend to support the caregiver’s responsibilities. Concerning the help provided to the cared-for person, this was mainly focused on keeping them company (n = 112, 81%), providing emotional support (n = 100, 73%), and helping them to feel safe (n = 95, 69%). However, help was also registered in regard to instrumental daily living activities, particularly with shopping needs (n = 64, 46%), cleaning (n = 52, 38%), and going to clinical appointments (n = 51, 37%). At the level of basic daily living, support with activities such as helping to get up and lay down in bed (n = 48, 35%), helping to sit down and get up from the chair (n = 43, 31%), feeding supervision (n = 39, 28%), and dressing (n = 39, 28%) are also mentioned.

### 3.7. Professional and Formal Support for Care Provided by the Caregiver

Professional support, considered in this paper as that which NGOs, parishes, and community resources provide, reaches approximately 39% (n = 120) of caregivers. Of those, one-quarter benefitted from these resources for more than 15 h per week (*M* = 14,4; *SD* = 27,0). Nine out of ten carers paid for this support (n = 102, 90%).

In respect to the care provided by these professionals, their support was predominantly allocated to basic daily living activities, such as dressing (n = 59, 52%), shower/bath (n = 52, 46%), bed/bath (n = 45, 40%), changing of diapers for urinary (n = 51, 45%) and fecal (n = 44, 39%) incontinence, and combing hair (n = 46, 41%). Support in instrumental daily living activities such as preparing meals (n = 35, 31%), preparing and administering medication (n = 21, 19%), and house cleaning (n = 19, 17%) were also described. Emotional support was only a concern for less than one-third of these professionals (n = 35, 31%). 

Approximately one quarter (n = 75, 26%) of the caregivers counted on support from formal private services in their care provision, with 75% stating that they benefitted from this help for more than 6 h per week (n = 55). Their contribution to the provision of care mostly occurred at the level of basic and instrumental daily living activities, similarly to the results reported for professional support—dressing (n = 42, 56%), house cleaning (n = 42, 56%), and meal preparation (n = 37, 49%).

### 3.8. Knowledge of Caregivers Regarding Community Resources

**Figure 1:**
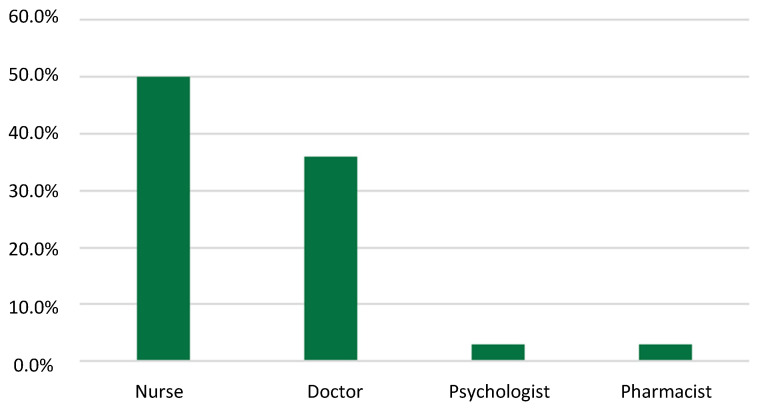
Health professionals’ contributions to the development of knowledge about community resources.

With respect to the knowledge of the caregiver regarding community resources, approximately three caregivers out of five did not know about them (n = 199, 64%). When this knowledge was present, it was mainly through the activity of social workers (n = 60, 54%) allocated to social institutions, hospitals, and parishes. Health professionals’ contributions to this knowledge were also significant to 44% of the caregivers (n = 48), of which half highlighted the nurse’s role in the process (n = 18), as Figure 1 shows. Internet (n = 18, 16%), television (n = 6, 5%), and associations/NGOs (n = 7, 2%) were also indicated as sources of knowledge.

Concerning the question of how community resources influenced caring activities, support groups, leaflets, videos, and manuals were less frequently identified by caregivers as support strategies. Two out of five (n = 130, 41%) admitted that they had never reached out for any support to care for a person. However, 30% had already sought help from a social institution (n = 92). Family, friends, coworkers, and others were also mentioned by a minority of our sample (n = 12, 4%).

Health services appeared to have significant importance as a support mechanism for caregivers. Approximately 30% (n = 94) relied on these institutions to help the cared-for person, mainly primary health care (n = 62, 75%), hospitals (n = 14, 17%), private institutions (n = 14, 17%), and the tertiary sector of care (n = 7, 8%). However, when caregivers faced a situation they did not know how to handle, this demand rose by about 130%: health professionals were the most frequently sought help resource (n = 222, 70%), particularly doctors (n = 133, 68%) and nurses (n = 92, 47%).

**Figure 2:**
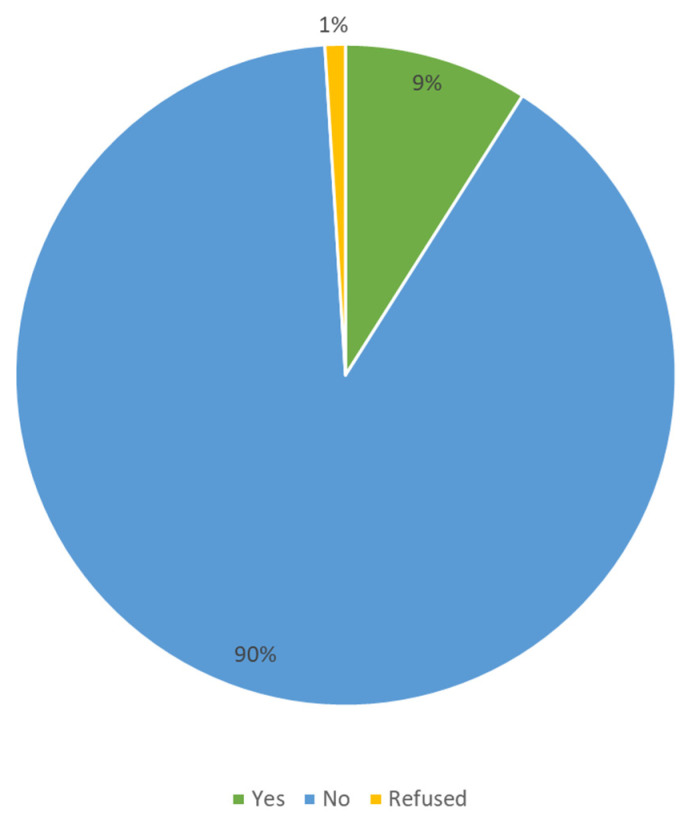
Caregiver access to support programs.

Regarding caregiver support programs, our study indicated that approximately 90% of the caregivers (n = 260) never had access to a program designed for this purpose (Figure 2). Programs with caregiver access and participation are summarized in Table 4.

### 3.9. Caregivers’ Rights and Duties

The law establishing the Statute of the Informal Caregiver, in which caregivers’ rights and duties are defined, was known about by 35% of the carers (n = 108). However, this knowledge was mainly based on the information circulating in the media and social networks. About 79% (n = 242) did not know how to be acknowledged as a caregiver by the appropriate official entity. Only fifteen caregivers (5%) in this study admitted to having benefited from their rights. Those who did not have that opportunity stated reasons such as problems with the acknowledgment system (n = 15, 5%) and a lack of knowledge (n = 8, 3%), among others.

Nevertheless, about one out of four caregivers claimed to know their rights (n = 75, 24%), mostly through television (n = 26, 36%), health professionals (n = 12, 17%), and social services (n = 15, 21%), although only a few provided suggestions regarding what should be optimized in this law, e.g., financial support for informal caregivers with professional activities (n = 5, 17%) and short-term caregiver replacement mechanisms (n = 5, 17%), among others.

With respect to the duties of the caregiver, two out of five affirmed that they knew these duties (n = 120, 40%). Care delivery with professional support from the health and social sector was the most identified duty (n = 53, 44%), followed by monitoring the cared-for person’s well-being (n = 35, 29%) and the satisfaction of basic and instrumental daily activities (n = 28, 23%).

Regarding this topic, participants in this study indicated that they sought help regarding the rights and duties of the caregiver in social services (n = 86, 27%), mainly in the case of those associated with the social and health sector and in healthcare facilities with health professionals (n = 73, 23%), as well as family (n = 24, 7%) and municipalities (n = 16, 5%).

The law establishing the right of an incapacitated person or their proxy to require guardianship from a ‘Responsible Adult’ (in Portugal, called “Lei do Maior Acompanhado”) was unknown to 81% of the caregivers (n = 233).

### 3.10. The Health of the Caregiver

Generally, carers indicated that they felt satisfied with their health level (n = 103, 40%), although approximately three out of ten did not feel satisfied or unsatisfied (n = 75, 29%). Regarding health follow-up, about 30% of caregivers received a home visit from a health professional in 2020 (n = 77). In 86% of the visits, these were performed by a nurse (n = 66), although other professionals were also identified, such as doctors (n = 34, 44%), physiotherapists (n = 21, 27%), and occupational therapists (n = 1, 1%), among others.

### 3.11. Impact of the COVID-19 Pandemic on Caregivers

The COVID-19 pandemic was noted as a factor affecting the caregiving activity by more than half of the respondents (n = 147, 56%), mainly due to social isolation (n = 58, 40%), the adoption of infection control measures (n = 57, 39%), the low availability of support resources to the caregiver and the cared-for person (n = 34, 23%), and the deterioration of the caregiver’s health status (n = 20, 14%). In seventeen cases (12%), the pandemic contributed to a rise in the need for care, having consequences on caregivers’ performance (n = 16, 11%) and in their professional context (n = 11, 8%).

## 4. Discussion

The results fulfilled the aim of this article, as they provide a local profile of informal caregivers’ characteristics, health, and social status, as well as revealing their needs and concerns, which were in alignment with the globally expressed needs of these groups of people caring for those in need. In this study, we verified several similarities between our data and other studies related to this topic and several opportunities for reflection regarding how to better support informal caregivers. To better organize the discussion, this chapter is written through the lens of the Model of Carer Stress and Burden [24].

### 4.1. Background and Contextual Factors

Regarding background and contextual factors, sociodemographic profiles of the cared-for person and the caregiver were the most relevant data for the outcome of the caregiving relationship in this study.

The reported profile of the person in need of care is similar to the findings of Ribeiro et al. [39] regarding sex, indicating that women were the most frequent care recipients (72%). However, some studies have shown that the sex distribution is more balanced [2,27]. The cared-for person was, on average, 80 years old, which is in alignment with the data described by Eurocarers and IRCCS-INRCA [27], which identified Portugal as the country with the oldest cared-for people in the European Union.

Regarding the caregiver, the female sex was identified as the most common in this profile, which also finds an echo in other studies [14,28,40]. In this sample, the age of the carer was, on average, 62 years old, which is in alignment with the interval proposed by the Embracing Carers platform—between 45 and 75 years old.

The carer’s civil status was also similar to what has been reported in the literature [39,41], with ‘married’ identified most frequently. Concerning the financial situation, most evidence indicates that carers’ financial situations are more problematic than what is reflected in the present study. Teixeira et al. and the platform Embracing Carers referred to 42% of caregivers as being in a low-income situation [28,40]. In this study, the majority of carers were retired, which may have influenced the low income of three out of ten carers. Although a significant portion of participants was effectively working, negotiation between the labor context and caregiving activities appeared to be a challenge in some cases, given that 18% of carers included in this study had to retire to provide care to a person in need.

Cohabitation was a reality for 63% of the caregivers in our sample, which is comparable with the results of a national study by Carvalho et al. [41]. This may be one of the reasons why a high amount of time spent on the caregiving role was reported, particularly in the case of almost one-quarter of the caregivers, who spent 24 h a day in a caregiving role. This fact can significantly hamper the balance between personal or professional life and the caregiving role, justifying why this topic should be a priority for the development of public policy, such as amplifying access to formal care.

In terms of kinship relationships, sons and daughters were the most common caregivers, and a large proportion of these had performed this role for over a year, subscribing to similar results reported by Eurocarers and IRCCS-INRCA [27].

### 4.2. Primary Stressors

In terms of primary stressors related to the caregiving activity itself, patient characteristics played a major role. The level of dependence found in care receivers in this study was severe, which overlaps with findings from other studies, possibly explaining why the caregivers’ main focus was on providing support for basic and instrumental daily living activities. 

Concerning the care situation, the main area where caregivers were required to help was instrumental in daily living activities, which is in accordance with the published data [16]. Emotional support, security, and management tasks are also reflected in the literature [30]. The nature of these tasks underscores the need for education for caregivers regarding how to provide high-quality and secure care to the person in a state of dependence, as well as the need to support them in their role, given the complexity of these activities and their potential burden. A study conducted recently in the United States uncovered results in line with this conclusion, indicating that caregivers who were responsible for performing complex medical or nursing tasks were more likely to develop emotional stress and physical strain [42].

The number of cared-for persons per caregiver was in agreement with the results from the survey of Eurocarers and IRCCS-INRCA, in which 78% of the caregivers also cared for one person [27]. However, the fact that 11% of the caregivers in this study were responsible for caring for more than one person raises concerns about the future, namely, about the resources available to support these caregivers and to promote a balance between their personal and professional life and caregiving activities.

### 4.3. Secondary Stressors

Maintaining a balance between one’s personal and professional life is recognized in the scientific literature as a universal challenge for caregivers. In this study, the results of our survey frequently identified COVID-19 as a relevant secondary stressor because the pandemic situation continued during the study development process. Several studies have called for attention to the particularly negative impact of this pandemic on caregivers. Eurocarers and IRCCS-INRCA identified a significant impact on social relationships, quality of life, and physical or mental health [27], whereas Sousa et al. reported a decrease in caregivers’ time and an increase in fatigue [43]. An additional burden was also mentioned in this regard, along with the need for support measures to assist carers in facing this problem [44].

Our results identified several other factors with impacts on the caregiver, such as the adoption of infection control measures, a decrease in formal care structures, and changes in the availability of support programs, as well as differences in their performance as a carer and their work context. This panel may help in the definition of immediate support measures and the development of a strategy for future similar situations.

However, in addition to these findings, the fact that caregivers were, in general, satisfied with their health is an interesting perspective, possibly supporting the Healthy Caregiver hypothesis by Roth et al. [45]. This was a populational study that stated that some caregivers showed positive outcomes of caregiving and, therefore, demonstrated that it is possible to avoid these stressors to provide good conditions for the provision of care.

### 4.4. Appraisal

Concerning the appraisal of caregivers’ capabilities to perform tasks targeting the needs of the cared-for person, most of them found themselves capable of accomplishing this task; nevertheless, more than one-third assumed that they needed help caring for the person in need. This need deserves to be valued and discussed by responsible organizations, as it may affect the quality of care and dignity of the cared-for person.

Apart from all the difficulties and needs identified, in regard to the reason or motivation to provide care, most caregivers indicated ‘love’ or ‘affection’ as the main reason to deliver care. Such a result is an almost new trend, given that there appears to be little evidence on this specific matter, only equivalent to the perspective of Hedler et al., who conceived of love as the center of a caring social representation [46].

### 4.5. Moderators (Exacerbating and Moderating Factors)

Informal caregivers, as the closest persons to those in need of care, have deep and concrete knowledge about what should be optimized to ensure dignity and the best possible quality of life for both people involved. Therefore, considering caregivers’ needs and their suggestions as a contribution to decision-making and policy development is key to ensuring a tailored long-term care system from the local to the global level.

The proportion of caregivers who count on help from family or friends in a secondary caregiver role varies between 50% and 65%, according to the available literature [27,39], and our findings are in line with this figure. However, the lack of family or friend support in more than half of the cases should raise a concern, as it may exacerbate feelings of overload [40].

The low percentage of caregivers with access to support programs in our study also raises a major concern. The presence of community support can also be a moderating factor in decreasing the occurrence of inadequacy in the caregiver role. According to Clemmensen et al. [47], in a Danish study about measures to support informal carers caring for a person with dementia, it is important to consider the existence of interventions targeting carers’ personal needs and the process of becoming a carer, as well as the provision of care and associated knowledge.

The need for the acknowledgment of caregivers is also a common point of discussion, as Courtin et al. indicate that most countries in the EU do not present adequate systems of identification and support for caregivers [16]. Zigante states that the family’s accountability in providing care to the cared-for person is common practice, particularly in southeastern Europe [6], and our results are aligned with this perspective. In this study, the lack of knowledge among a high proportion of participants regarding how to be acknowledged as a caregiver shows the need to improve acknowledgment systems and their dissemination through the community.

Spasova et al. suggested important measures to optimize support to informal caregivers, such as improving caregiver rest modalities, developing more support systems for caregivers, and better balancing between caregiving and professional activities [17]. The suggestions made by this study’s participants went beyond these, underlining the optimization of bureaucracy and the acknowledgment of caregivers providing care to people with mental disorders or psychosocial vulnerabilities.

### 4.6. Outcomes and Implications for Clinical Practice and Research

The importance of caregivers has been progressively recognized as part of the global agenda. In the last *Health at a Glance* report, the OECD assumed that informal and family caregivers were the main source of care providers ensuring long-term care [48]. They suggested the existence of a trade-off between formal and informal care as few people provide the latter type of care in countries with adequate long-term care systems [48].

More recently, the European Commission has also launched the discussion about an EU strategy for care, stating the importance of ensuring caregivers’ and cared-for persons’ access to long-term care and promoting a balance between the personal life of the person and their caregiving role [49]. This initiative follows the lead of the chronic need in our health system to provide high-quality long-term care.

Furthermore, involvement by informal and family caregivers is crucial to the success of any strategy in development, given their expertise and proper knowledge about the matters in the discussion. Data collected in this study show how all the concepts related to informal caregivers display themselves as pluridimensional, demanding an equally interdisciplinary response. However, this appears to continue to be a significant need, given the scope of needs portrayed in this study.

Nurses can act as key actors in this response, given their proximity to caregiving contexts and the supporting role they provide to carers and people who they care for. Conceiving a background where any action is intended to increase collaboration between individuals, families, and communities, nursing interventions have the potential to influence real lives, including caring and supporting caregivers and the people they care for while also promoting health and preventing disease [50].

The results of a recent study regarding educational interventions by nurses targeting an elderly population at home were in line with this perspective, indicating that encouraging communication, improving counseling and health education, as well as optimizing access to health information are significant strategies when discussing support for informal caregivers [51]. Another study from the United States mentioned the need to improve communication between formal and informal caregivers, specifically regarding the adequacy of medical language to increase people’s knowledge [52].

The operationalization of nursing’s contribution to this problem also depends on knowledge about the daily experiences and challenges that caregivers face. In Poland, a study conducted in 2019 on the influence of long-term care nursing on satisfaction, workload stress, and support demonstrated a positive relationship between nursing intervention and caregiver situations, improving the quality of their delivery of care [53].

Furthermore, national and regional profiles are of the utmost importance in assessing general measures and regimes. However, local profiles can be taken to a different level, connecting people’s needs to the specifics of a certain context, potentially increasing their understanding and solving their health and social problems. In this approach, intersectoral collaboration is crucial to developing a caregivers’ functional support network, which is in line with a recent European study that underlined the role of collaborative efforts and multifaceted interventions in enhancing caregivers’ resilience [54]. 

As a nurse-led project, in this study, we aimed to highlight people’s needs and inspire policy changes and tailored interventions to better support caregivers. Establishing a partnership with several institutions and the possibility of producing a county profile to assess local decision-making were the major gains of this study while acknowledging the certainty that there is still a lot to be accomplished in the future. Nursing interventions can assume a leading role in promoting health literacy, providing emotional support and positive communication, and creating a connecting point between healthcare workers and informal caregivers. More evidence is needed to support decision-making and awareness regarding the importance of informal caregivers and their contribution to the health system has to be a pivotal priority.

### 4.7. Limitations

The limitations of this study are associated, on the one hand, with its characteristics. Given that data collection was mainly conducted in a digital and, thus, impersonal manner, control and follow-up of some indicated contacts was relatively difficult, resulting in non-inclusion (n = 47). Some participants also identified this digital approach as a disadvantage since the interview was developed more often through a phone call than in person.

Another aspect that can be configured as a limitation is the lack of points of comparison in the available scientific literature. This is related to the pioneering characteristics of some indicators studied in this survey, e.g., evaluations of caregivers’ professional backgrounds and dependence related to the cared-for person, among others. The fact that caregivers were included without regard for the age of the cared-for person can also be configured as a limitation, given that there are differences between caring for an older adult and caring for a child with a chronic condition.

In addition, the absence of a validated measure to assess the health of the caregiver decreases the level of comparison possible with similar evidence, even though the results concerning this topic are valid for the definition of a caregiver profile. Caregivers’ self-reporting of all medical and demographic information is also a limitation of this study.

## 5. Conclusions

Through this profile of informal caregivers in Lisbon, we identified several matters of concern and unsatisfied needs related to this social group. Raising awareness about caregivers, supporting their role, and reflecting on how the relevant operational, social, and political systems can be optimized is crucial to promote better conditions for informal care and maximize the quality of life of the caregiver and the cared-for person. Investment in future research related to this matter is needed to provide deeper knowledge about caregivers and to develop meaningful interventions targeting significant health needs.

## Figures and Tables

**Table 1:** Inclusion and exclusion criteria.

Inclusion Criteria	Exclusion Criteria
Informal caregiver aged 18 years old or above; Informal caregiver providing care for a care receiver living in Lisbon county; Informal caregiver providing care for a care receiver in a home setting; Informal caregiver providing care to a care receiver in, at least, one dimension of self-care.	Informal caregiver aged 17 years old or below; Informal caregiver providing care to a care receiver living outside the Lisbon county or in other country; Informal caregiver providing care to a care receiver in community settings or residential facilities; Formal caregiver providing care to a care receiver. Informal caregivers providing care to a child without a chronic or disability condition.

**Table 2:** Caregivers’ support for the cared-for person’s basic daily living activities.

Basic Daily Living Activities					
	n	%		n	%
Combing	136	45%	Eating/Feeding supervision	158	52%
Brushing teeth	96	32%	Helping to walk	101	33%
Shaving	32	11%	Helping to get up and lay down in bed	124	41%
Shower bath	108	36%	Helping to sit and get up from the chair	114	38%
Bed bath	60	20%	Helping to position the person in bed	92	31%
Choice of clothes	159	53%	Helping to walk up and downstairs	94	31%
Dressing	168	56%	Change diapers (fecal incontinence)	98	33%
Helping to set food on the plate	123	41%	Change diapers (urinary incontinence)	123	41%
Eating/Feeding	75	25%	Helping the person to use the bathroom	93	31%

**Table 3:** Caregivers’ support for the cared-for person’s instrumental daily living activities.

Instrumental Daily Living Activities					
	n	%		n	%
Answer telephone	110	36%	Go to clinical appointments	236	78%
Shopping	264	87%	Prepare and administer medication	181	60%
Preparing meals	223	74%	Prepare and remind medication self-administration	129	43%
House cleaning	211	70%	Supervision (only) of medication self-administration	65	22%
Laundry	210	70%	Manage finances and legal matters	245	81%
Provide transport	186	62%			

**Table 4:** Support programs or services with caregiver access and participation.

Caregiver Support Programs and/or Services	n	%
Structured program on disease information, resources, and services	11	3.2%
Structured training program about symptom management and care delivery	7	2.0%
Structured training program about the needs and problems of the caregiver	9	2.6%
Self-assistance group	12	3.5%
Support group	15	4.4%
Psychotherapy	7	2.0%
Regular visit from a volunteer that enables free time to go out	0	0.0%
Regular visit from a volunteer to vent or talk	0	0.0%
Daycare center for the person with dependence	3	0.9%
Temporary admission into a healthcare facility for caregiver rest/vacation	7	2.0%

## Data Availability

The data presented in this study are available on request from the corresponding author. The data are not publicly available due to privacy reasons.
